# RNA-Seq with a novel glabrous-ZM24*fl* reveals some key lncRNAs and the associated targets in fiber initiation of cotton

**DOI:** 10.1186/s12870-022-03444-9

**Published:** 2022-02-03

**Authors:** Xianyan Zou, Faiza Ali, Shuangxia Jin, Fuguang Li, Zhi Wang

**Affiliations:** 1 State Key Laboratory of Cotton Biology, Key Laboratory of Biological and Genetic Breeding of Cotton, Institute of Cotton Research, Chinese Academy of Agricultural Sciences, Anyang, 455000 China; 2 National Key Laboratory of Crop Genetic Improvement, Huazhong Agricultural University, Wuhan, 430070 China; 3 Zhengzhou Research Base, State Key Laboratory of Cotton Biology, School of Agricultural Sciences, Zhengzhou University, Zhengzhou, 450001 China

**Keywords:** Glabrous cotton, RNA-Seq, Fiber initiation, Fiber development, LncRNA, Transcription factor

## Abstract

**Background:**

Cotton fiber is an important natural resource for textile industry and an excellent model for cell biology study. Application of glabrous mutant cotton and high-throughput sequencing facilitates the identification of key genes and pathways for fiber development and cell differentiation and elongation. LncRNA is a type of ncRNA with more than 200 nt in length and functions in the ways of chromatin modification, transcriptional and post-transcriptional modification, and so on. However, the detailed lncRNA and associated mechanisms for fiber initiation are still unclear in cotton.

**Results:**

In this study, we used a novel glabrous mutant ZM24*fl*, which is endowed with higher somatic embryogenesis, and functions as an ideal receptor for cotton genetic transformation. Combined with the high-throughput sequencing, fatty acid pathway and some transcription factors such as MYB, ERF and bHLH families were identified the important roles in fiber initiation; furthermore, 3,288 lncRNAs were identified, and some differentially expressed lncRNAs were also analyzed. From the comparisons of ZM24_0 DPA vs ZM24_-2 DPA and *fl*_0 DPA vs ZM24_0 DPA, one common lncRNA MSTRG 2723.1 was found that function upstream of fatty acid metabolism, MBY25-mediating pathway, and pectin metabolism to regulate fiber initiation. In addition, other lncRNAs MSTRG 3390.1, MSTRG 48719.1, and MSTRG 31176.1 were also showed potential important roles in fiber development; and the co-expression analysis between lncRNAs and targets showed the distinct models of different lncRNAs and complicated interaction between lncRNAs in fiber development of cotton.

**Conclusions:**

From the above results, a key lncRNA MSTRG 2723.1 was identified that might mediate some key genes transcription of fatty acid metabolism, MYB25-mediating pathway, and pectin metabolism to regulate fiber initiation of ZM24 cultivar. Co-expression analysis implied that some other important lncRNAs (e.g., MSTRG 3390.1, MSTRG 48719.1, and MSTRG 31176.1) were also showed the different regulatory model and interaction between them, which proposes some valuable clues for the lncRNAs associated mechanisms in fiber development.

**Supplementary Information:**

The online version contains supplementary material available at 10.1186/s12870-022-03444-9.

## Introduction

Cotton (*Gossypium* spp.) is one of the most important cash crops in the world because its main product fiber is the important natural source for the textile industry. In the four cultivars of *Gossypium* genus (*G.hirsutum*, *G. barbadense*, *G. arboreum*, and *G.raimondii*), *G hirsutum* (upland cotton) is the most widely planted due to its high yields and adaptability [42]. The period of cotton fiber development has been classified into four stages: initiation, elongation, secondary cell wall deposition, and maturity of fiber [12]. The first two stages could determine the number and length of fibers, further affecting fiber yields. Consequently, many studies have been documented to explore the underlying genetic mechanisms related to fiber initiation and elongation, contributing to cotton production improvement [16, 17, 19, 29, 65].

Cotton mutants with fibreless, fuzzless, and lintless are good materials for studying the mechanism of fiber initiation development. With the auxin and gibberellin (GA) application in two fibreless mutants of Asian cotton *in vitro* culture, it showed that fiber cells differentiated from ovule epidermis at a temperature lower than 30 degrees, but not above 32 degrees, which indicated the important roles of auxin and GA in fiber development promotion at some specific conditions [2]. SNPs comparison obtained by RNA-Seqs showed that glabrous mutant Xu142*fl* may be the progeny of *G. barbadense*. Based on the F_2_ and BC_1_ population between TM-1 and Xu142*fl*, the *Li*_*3*_ gene encoding an MYB-MIXTA-like transcription factor was mapped and adjacent to *MYB25-like* in the D12 chromosome [60]. The inheritance evaluation of fuzzless seed in segregation population suggested that the interaction of three loci (*N*_*1*_, *n*_*2*_ and *n*_*3*_) contributed to fuzzless seed [48], among which two loci, *N*_1_ and *n*_2_, located on a pair of homologous chromosomes A12/D12 [6]. The plants of *N*_*1*_*N*_*1*_ homozygous and *N*_*1*_*n*_*1*_ heterozygous produced fuzzless seeds [48]. The *n*_*3*_ locus that could produce the fibreless seed was identified by genetic analysis of cross progeny between *N*_*1*_*N*_*1*_ and *n*_*2*_*n*_*2*_ *[*48*]*. The fourth locus, named *n*^*t*^_*4*_${n}_t^4$, was identified from ethyl methanesulfonate (EMS) induced mutation analysis, whose homozygous seed exhibited a partially naked phenotype [3]. All these fiber development defect mutants provide suitable materials for fiber development study.

With the advantage of Next Generation Sequencing (NGS), RNA-Seq as one of the NGS has been widely used to reveal expressions of genes and transcripts, among which some transcripts have been identified as non-coding RNA (ncRNA) because of their limitation of coding proteins. NcRNA includes microRNAs (miRNAs), long non-coding RNAs (lncRNAs), and so on, which have emerged as key regulators of gene expression through their direct and indirect actions on chromatin [23–25]. In *Oryza sativa*, 1,254 differentially expressed lncRNAs (DELs) were identified from BIL progenies [26]. Another RNA-Seq showed that 328 of 444 DELs were associated with meiosis and the low fertility in autotetraploid rice [27]. The lncRNAs were also involved in abiotic stress such as drought and re-watering in *Brassica napus* [46], and osmotic and salt stress in *Medicago truncatula* [53]. The differences in genes expressions and regulations between fibreless mutants and wild-type have been investigated using omics methods [14, 28, 45, 51]. With fiberless mutant Xu142*fl* and its counterpart Xu142, a previous comparative small RNAome analysis uncovered a possible network of fiber initiation-related miRNAs in cotton ovules, which comprises seven miRNAs expressed in cotton ovules, and each of them bears functional specific targets [51]. Another work showed that 54 miRNAs are differentially expressed in fiber initiation between Xu142*fl* and its wild-type, which are potentially targeted to TFs such as MYB, auxin response factor, and Leucine repeat receptor [45]. Using multi-omics, the differentially expressed genes (1,953), proteins (187), and phosphoproteins (131) were identified by the comparison of Xu142 and Xu142*fl* [28]. Genetic markers including 302 SNPs for fiber development were also developed and validated based on a deep sequencing between Xu142 and Xu142*fl* [28]. In particular, a transcriptomic repertoire revealed that 645 and 651 lncRNAs were preferentially expressed in Xu142*fl* and Xu142, respectively. Further study showed that down-regulating two lncRNAs XLOC_545639 and XLOC_039050 in Xu142 *fl* increased the fiber initials on the ovules, while silencing XLOC_079089 in Xu142 shortened the fiber length [14], indicating the important and diverse roles of lncRNAs in fiber development.

LncRNA is a type of ncRNA with more than 200 nt in length and without protein-coding abilities [4, 70]. Lots of evidence have shown that lncRNAs could regulate genes functions in the ways of chromatin modification, transcriptional and post-transcriptional modification, etc. [32], through which the lncRNAs play vital roles in plant growth, development [67], and response to biotic [68] and abiotic stresses [24, 43, 62]. LncRNAs could be classified into long intergenic non-coding RNAs (lincRNAs), natural antisense non-coding RNAs (lncNAT), sense non-coding lncRNAs, and intronic lncRNAs according to their location in the genome [5, 31, 55]. In cotton fiber development, the detailed regulation mechanism of lncRNA is still ambiguous. In this research, we introduced a natural lintless-fuzzless (ZM24*fl*) mutant from zhongmiansuo24 (ZM24) cultivar, which is easy for transformation [8, 59]. Further, strand-specific transcriptome sequencing was conducted to reveal the differential expression profile of genes in the fiber initiation periods including lint and fuzz initiation stages. The lncRNAs/PC-genes pairs are identified and analyzed to explore the potential key lncRNAs and the corresponding targets contributing to the fiber development differences between the ZM24*fl* and ZM24.

## Results

ZM24 (*G. hirsutum*) is a high-quality commercial cotton cultivar bred by the Chinese Cotton Research Institute; this cultivar exhibits excellent somatic embryogenesis potential, which makes it an ideal receptor for cotton genetic transformation [59]. We identified a spontaneous mutation in ZM24 that resulted in the production of fuzzless and lintless seed and designated it as ZM24 fuzzless-lintless (ZM24*fl*), which would be indicated as *fl* in the following study*.*

### The difference of the fiber cell development between *fl* mutant and ZM24

Firstly, we observed the vegetative development of *fl* and ZM24; the results showed that the development of the organs and tissues such as branches, leaves, bolls, and epidermal hair of leaves, and stems is normal in *fl*, and similar with ZM24, except that the boll size is smaller in *fl* due to fiber development defects (Figure S1).

**Fig. 1:**
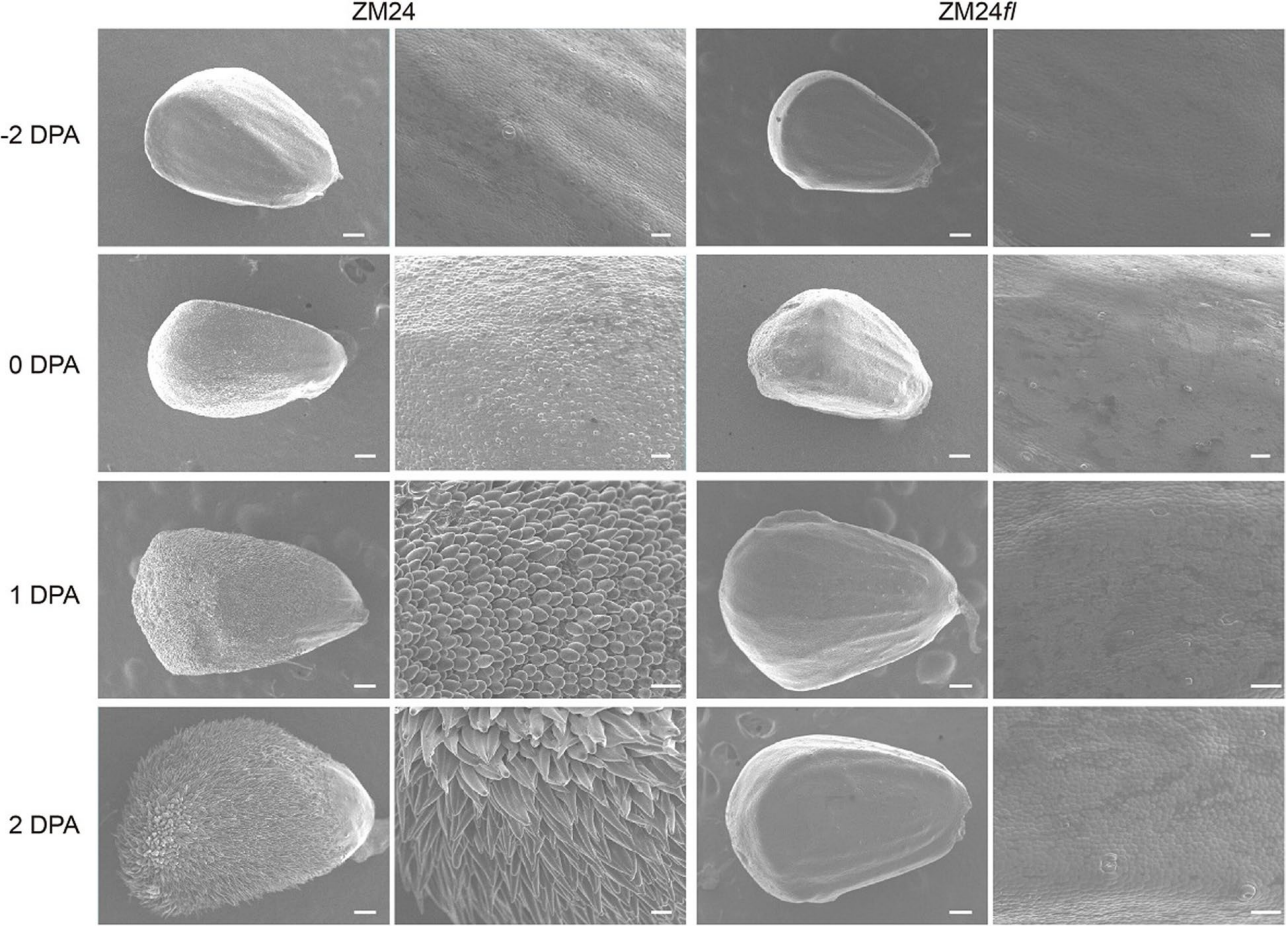
The microscope of ovules during fiber initiation stages in ZM24 and the *fl* mutant. The SEM photographs of ovules in ZM24 (left) and ZM24 *fl* (right) on -2, 0, 1 and 2 DPA. All ovules were taken from the same position of the bolls in a similar position on each plant. bars, 40 μm (magnified) and 150 μm

To reveal the difference of fiber development between *fl* and ZM24 during fiber initiation stages, ovules at -2 day-post-anthesis (DPA), 0 DPA, 1 DPA and 2 DPA from two lines were stripped for observation using scanning electron microscopy. The scanning results showed that there were no significant differences on the epidermis of -2 DPA ovules in both lines (Fig. 1). The epidermis of ovules from 0 to 2 DPA, which indicated the fiber initiation stage, presents the clear differences of fibrous protuberance between ZM24 and *fl*. The fibrous protuberance appeared on the epidermis of 0 DPA ovules in ZM24, and they slowly elongated over time. However, no fiber was observed on the epidermis of ovules of 0 DPA, 1 DPA, and 2 DPA from *fl* (Fig. 1), which indicated that *fl* is an excellent line for fiber cell initiation study.

### Identification and characterization of lncRNAs in cotton fiber initiation

**Fig. 2:**
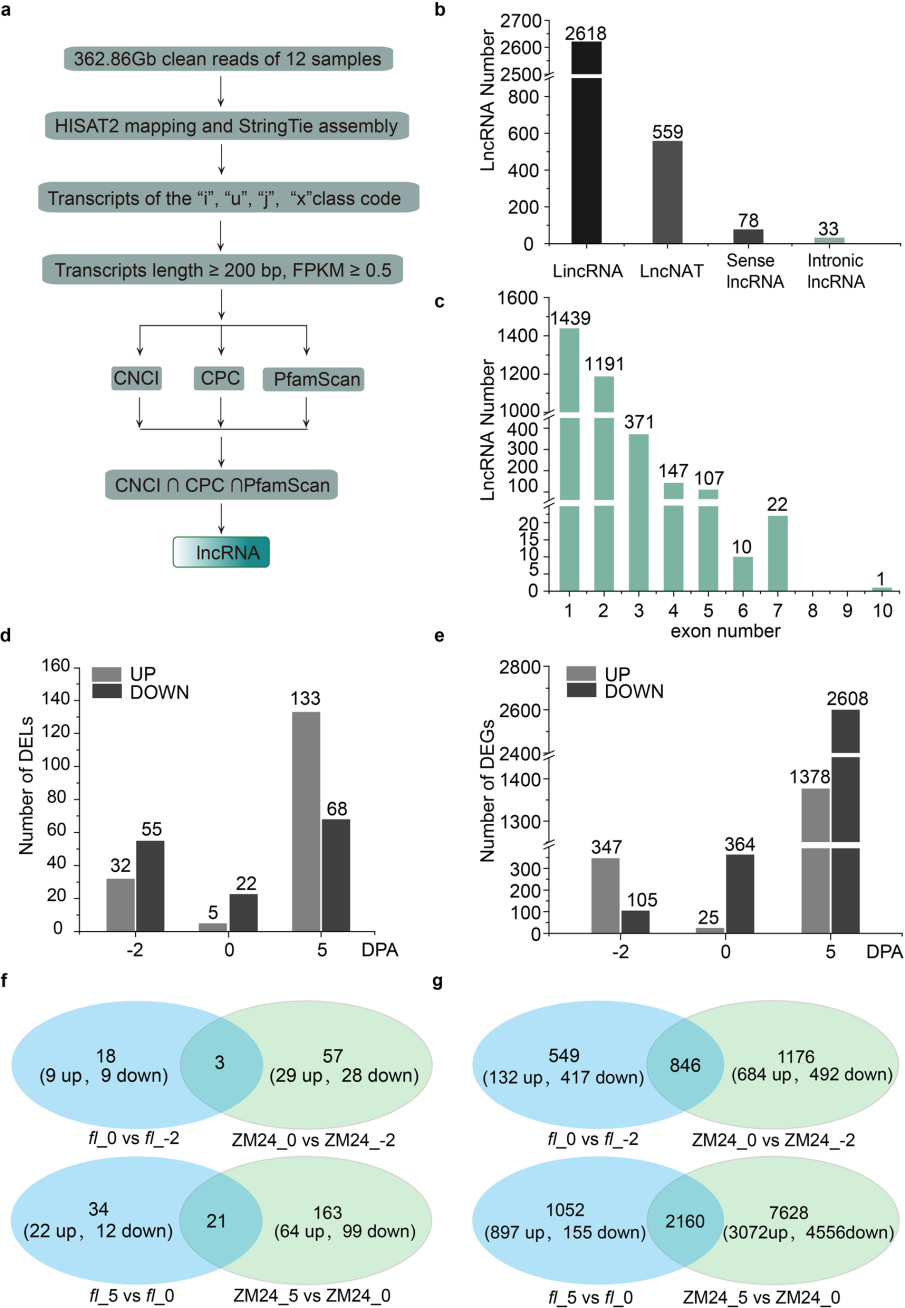
An integrative computational pipeline for the bioinformatics analysis and summary of DELs and DEGs in the RNA-Seq. **a** Bioinformatics methods for the identification of lncRNA. **b** The number of lincRNA, lncNAT, sense lncRNA, and ntronic lncRNA. **c** Exon number pattern of four kinds of lncRNA. **d** and **e** Number of differentially expressed lncRNAs and genes (up-and down-regulated) between *fl* and ZM24, respectively, in three time points (-2, 0, and 5 DPA) during fiber initiation. **f** and **g** Venn diagrams showing the common DELs and DEGs between comparisons of ‘0 DPA vs -2 DPA’ and ‘5 DPA vs 0 DPA’ of *fl* and ZM24, respectively. DELs and DEGs were identified with log_2_| (fold change)| > 1, FDR < 0.001

LncRNAs have been shown the important roles in fiber development [14]. To investigate the potent lncRNAs responsible for the fiber initiation, we performed strand-specific RNA sequencing on 6 ovule samples (-2 DPA, 0 DPA, and 5 DPA from ZM24 and *fl*) with twice replicates. A total of 362.86 Gb clean reads were obtained by removing low-quality reads, and all Q30 of RNA-Seq results are greater than 85%, indicating that clean reads were qualified for downstream analysis (Additional file 1). To assess the correlations between biological replicates, the Pearson correlation was calculated using FPKM assays and the r^2^ between two replicates of each material was above 0.88, indicating the high correlation and reliable data (Figure S2). An integrated pipeline (Fig. 2a) was used to identify lncRNA in these tissues (see details in materials and methods). Finally, 3,288 lncRNA transcripts were obtained, among which the lincRNAs (2,618), lncNATs (559), sense lncRNAs (78) and intronic lncRNAs (33) accounted for 79.6 %, 17 %, 2.4 %, and 1 %, respectively (Fig. 2b). The pattern of exons in lncRNA transcripts showed that single-exon lncRNAs represented the largest proportion, accounting for 43.8% (Fig. 2c). The physical positions and the class codes of lncRNAs were detailed in Additional file 2.

### Differentially expressed lncRNAs and protein-encoding genes between *fl* and ZM24

Furthermore, the clean reads were mapped to ZM24 genomes, and FPKM and counts of transcripts and genes were identified for further analysis with a threshold of |log_2_(FC)| > 1 and *p*-value < 0.05. In summary, a total of 371 differentially expressed lncRNAs (DELs) and 12,971 DEGs were identified in vertical (*fl* vs ZM24 at the same developmental stages) and horizontal (different development stages in the same line) comparisons, respectively. The FPKM values of the DELs and DEGs were shown in Additional files 3 and 4.

In detail, 87 (32 up-regulated, 55 down-regulated), 27 (5 up-regulated, 22 down-regulated), and 201 (133 up-regulated, 68 down-regulated) lncRNAs were differentially expressed in the -2, 0, and 5 DPA ovules of *fl* compared with that in ZM24, respectively (Fig. 2d), which indicated that many down-regulated lncRNAs in the -2 and 0 DPA ovules of *fl* genes play potential roles in fiber initiation. Correspondingly, there were 452 (347 up- and 105 down-regulated), 389 (25 up- and 364 down-regulated), and 3,986 DEGs (1,378 up- and 2,608 down-regulated) in *fl* compared to ZM24 at the three time points of fiber initiation stage, respectively (Fig. 2e), these results indicated that the lncRNAs might regulate target genes encoding proteins either negatively or positively.

To further investigate the DEGs between *fl* and ZM24 at different development stages, the DELs and DEGs of “0 DPA vs -2 DPA” and “5 DPA vs 0 DPA” were identified, and the Venn diagram established showed that 21 (10 up- and 11 down-regulated) and 60 (30 up- and 30 down-regulated) DELs were identified in *fl* and ZM24, respectively, in the comparison of 0_DPA vs -2 DPA, of which only 3 common DELs (1 up-regulated, 2 down-regulated)) were identified in both comparisons. Similarly, 218 DELs were identified in comparison of 5 DPA vs 0 DPA, in which, 34 and 163 DELs were unique to *fl* and ZM24, respectively. Interestingly, in the other 21 common DELs, 17 and 3 DELs were up- and down-regulated at 5 DPA compared to 0 DPA; only one DELs, MSTRG.3394.1, was down regulated in *fl*_5DPA compared with *fl*_0DPA, but up-regulated in that of ZM24. These results indicated that more lncRNA were involved in the fiber development than that in the ovule development. Consistently, a lot of DEGs were identified from the comparisons (Fig. 2g), suggesting that the dramatic transcription regulation differences between two lines might contribute to the development of the fiber in ZM24. These results proposed that the expression of genes including protein-encoding genes and lncRNA-encoding genes changed dramatically in the two lines, which may contribute to the formation of the glabrous seed of *fl*, however, the underlying mechanism is unclear.

### GO enrichment of DEGs

**Fig. 3:**
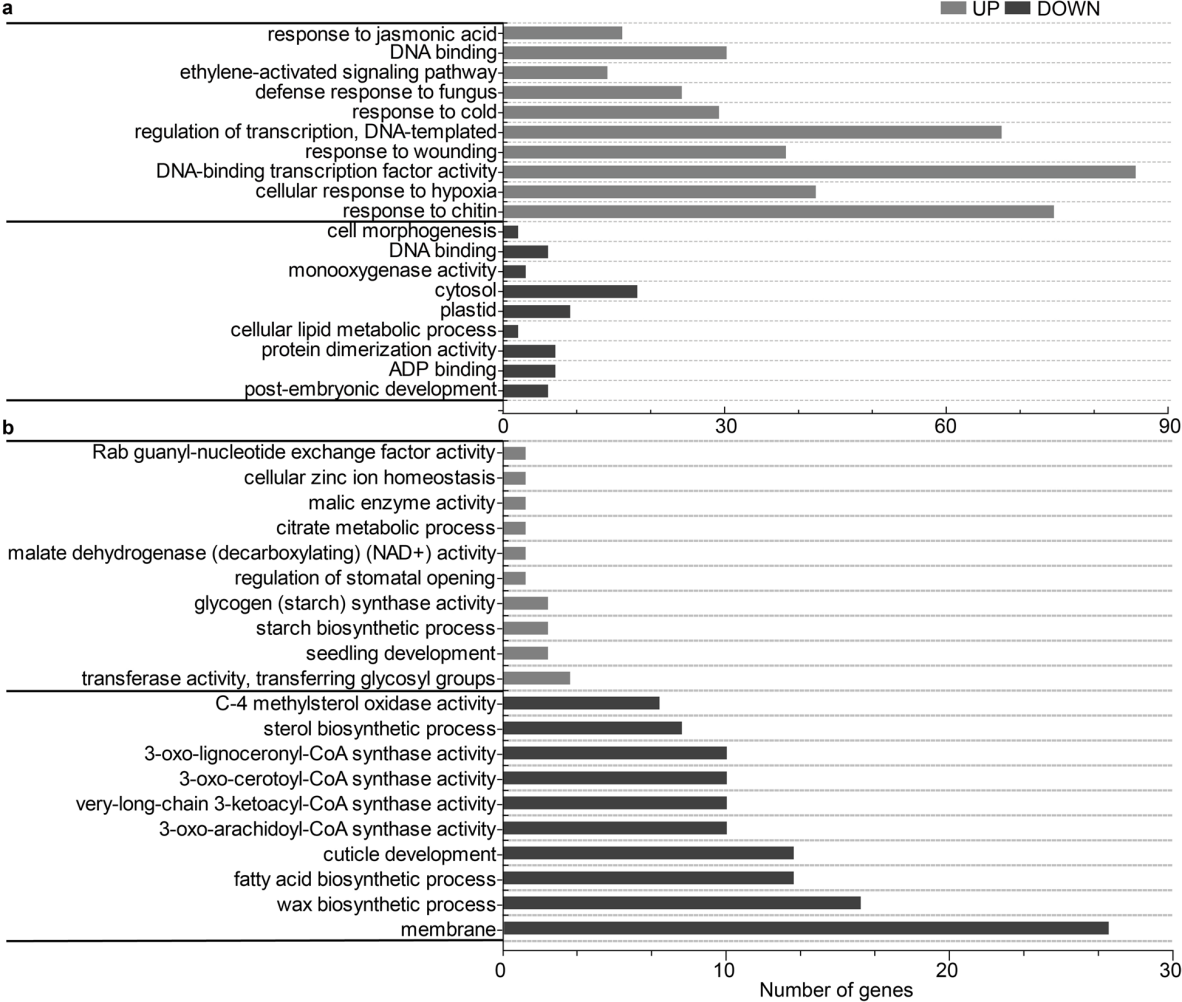
Gene ontology classifications of DEGs between *fl* vs ZM24 at -2 DPA and 0 PDA. The most highly enriched GO terms showed the 105 down- and 347 up-regulated genes in ovules of -2 DPA (**a**), and the 364 down- and 25 up-regulated genes in ovules of 0 DPA of ZM24 comparing with *fl* (**b**), respectively

To investigate the functions of DEGs between *fl* and ZM24 in fiber initiation and elongation, the GO enrichment analysis of DEGs was performed to explore the causal pathways or genes responsible for the phenotype differences in two lines. According to the results of GO enrichment, the most significant (corrected *p*-value < 0.05) classes were identified. On -2 DPA, the biological process of regulation activity and exogenous and endogenous signals such as cold (GO:0009409), fungus (GO:0050832), jasmonic acid (GO:0009753), and chitin (GO:0010200) were mainly enriched in up-regulated genes, whereas the down-regulated DEGs were enriched in the cellular process such as “cytosol” (GO:0005829), “plastid” (GO:0009536), “post-embryonic development” (GO:0009791) in *fl* compared with ZM24 (Fig. 3a). On the day of flowering, the down-regulated genes were enriched in terms of “wax biosynthetic process” (GO:0010025), “cuticle development” (GO:0042335), “3-oxo-lignoceronyl-CoA synthase activity” (GO:0102338), “3-oxo-cerotoyl-CoA synthase activity” (GO:0102337), and “fatty acid biosynthetic process” (GO:0006633), which were related to lipid metabolism (Fig. 3b), indicating that cell wall plasticity regulated by lipid metabolism might play important roles in fiber cell initiation. On 5 DPA, the earlier fiber elongation stage, the DEGs of two lines exhibited more significant functions in cellular components (Fig. S3). For example, the down-regulated genes in “*fl*_5 vs ZM24_5” comparison was mostly enriched in terms of “plasma membrane” (GO:0005886), “cytoplasm” (GO:0005737), “integral component of membrane” (GO:0016021), etc., indicating that fiber development requires the expression and regulation of a large number of genes to bring cellular component effect.

### Identification of potential genes for fiber initiation

**Fig. 4:**
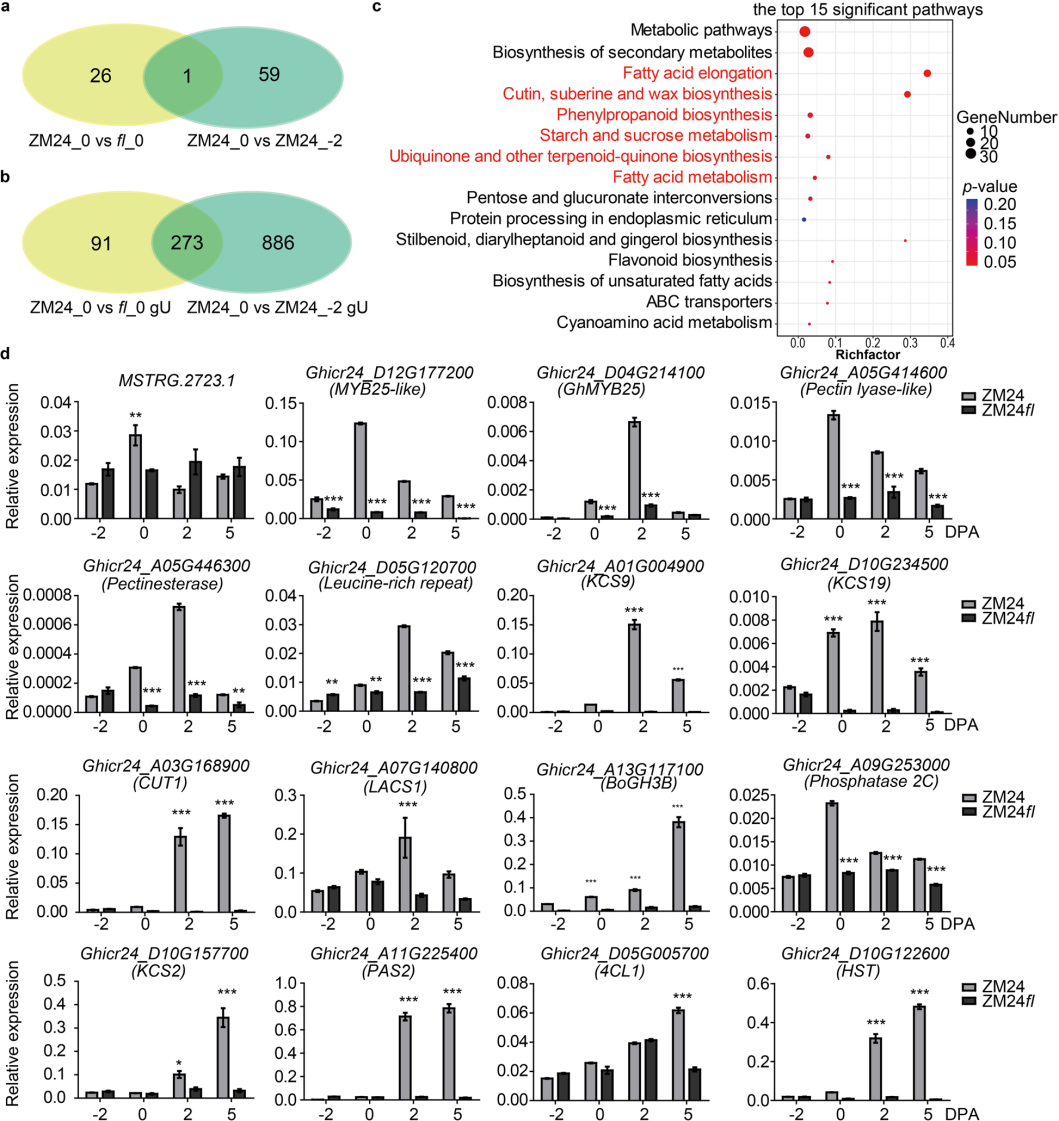
The potential lncRNAs and genes for fiber initiation. **a** and **b** Venn diagrams showed the common lncRNAs and genes in DELs and DEGs from comparisons of “ZM24_0 vs *fl*_0” and “ZM24_0 vs ZM24_-2”, respectively. gU represented the up-regulated genes. **c** The KEGG pathways of the commonly targeted genes of lncRNA and the common DEGs. **d** qPCR experiments confirmed the expression profiles of the common lncRNA (MSTRG.2723.1), its target genes, and some DEGs that enriched in fatty acid elongation and phenylpropanoid biosynthesis. The *GhHistone3* (AF024716) gene was used as a reference gene, and the data were shown as mean ± SD. The student’s *t*-test was used for the significance statistic

Generally, the phenotype is determined by genotype. Therefore, the reason why the *fl* exhibits a fibreless phenotype is mainly due to the differences in transcriptional regulation that happened during a few days before the flowering. To analyze and obtain genes sets related to fiber initiation, venn diagrams were conducted to show the common target between “ZM24_0 vs *fl*_0” and “ZM24_0 vs ZM24_-2”. Subsequently, one common DEL, MSTRG2723.1, was identified between “ZM24_0 vs *fl*_0” and “ZM24_0 vs ZM24_-2” (Fig. 4a), which showed significantly higher expression in ovules of ZM24_0 DPA compared with *fl*_0 DPA and ZM24_-2 DPA, and targeted to 36 DEGs (Additional files 3 and 5). Moreover, 273 common DEGs between the up-regulated genes in ZM24_0 vs *fl*_0 and ZM24_0 vs ZM24_-2 comparisons were identified (Fig. 4b). Hence, KEGG enrichment showed that these targeted genes and DEGs were significantly enriched in “fatty acid elongation” (12 genes), “cutin, suberine, and wax biosynthesis” (9 genes), “phenylpropanoid biosynthesis” (5 genes), “starch and sucrose metabolism” (4 genes), “ubiquinone and another terpenoid-quinone biosynthesis” (3 genes) and “fatty acid metabolism” (3 genes) pathways that possibly be related to fiber initiation (Fig. 4c). To validate the RNA-Seq results, the specific primers of the lncRNA MSTRG2723.1, and the randomly selected 11 genes from the targeted genes and DEGs were designed and used for Q-PCR. Relative expression levels of these genes in -2, 0, 2, and 5 DPA ovules were identified and consistent with the RNA-Seq. Of these, the transcription factor coding genes *GhMYB25-like* and *GhMYB25* that were positive to fiber initiation were down-regulated in *fl*. In addition, the genes that were annotated as pectin lyase-like, pectinesterase, and a leucine-rich repeat (LRR) were down-regulated in *fl* compared to ZM24. Furthermore, the encoding 3-ketoacyl-CoA synthase genes *Ghicr24_A01G004900* (*KCS9*) and *Ghicr24_D10G234500* (*KCS19*), targeted by MSTRG.2723.1, were significantly down-regulated in *fl* during the initiation stage, and enriched in the fatty acid elongation pathway (Fig. 4d). The down-regulated genes such as *CUT1* and *LONG CHAIN ACYL-COA SYNTHASE 1* (*LACS1*) are required for elongation of C24 very-long-chain fatty acid (VLCFA) with a function in wax production [33]. The gene *GhPAS2*, enriched in the fatty acid metabolism pathway, was also characterized as the enzyme that catalyzes VLCFA production [54]. The genes *4-COUMARATE-COA LIGASE 1* (*4CL1*) and *O-HYDROXYCINNAMOYLTRANSFERASE* (*HST*) that were down-regulated in *fl* and enriched in phenylpropanoid biosynthesis pathway, affected the lignin composition [13]. These results indicated that the potential targeted genes involved in VLCFA by lncRNAs may play critical roles in fiber initiation.

### Identification of genes encoding transcription factors from the DEGs

**Fig. 5:**
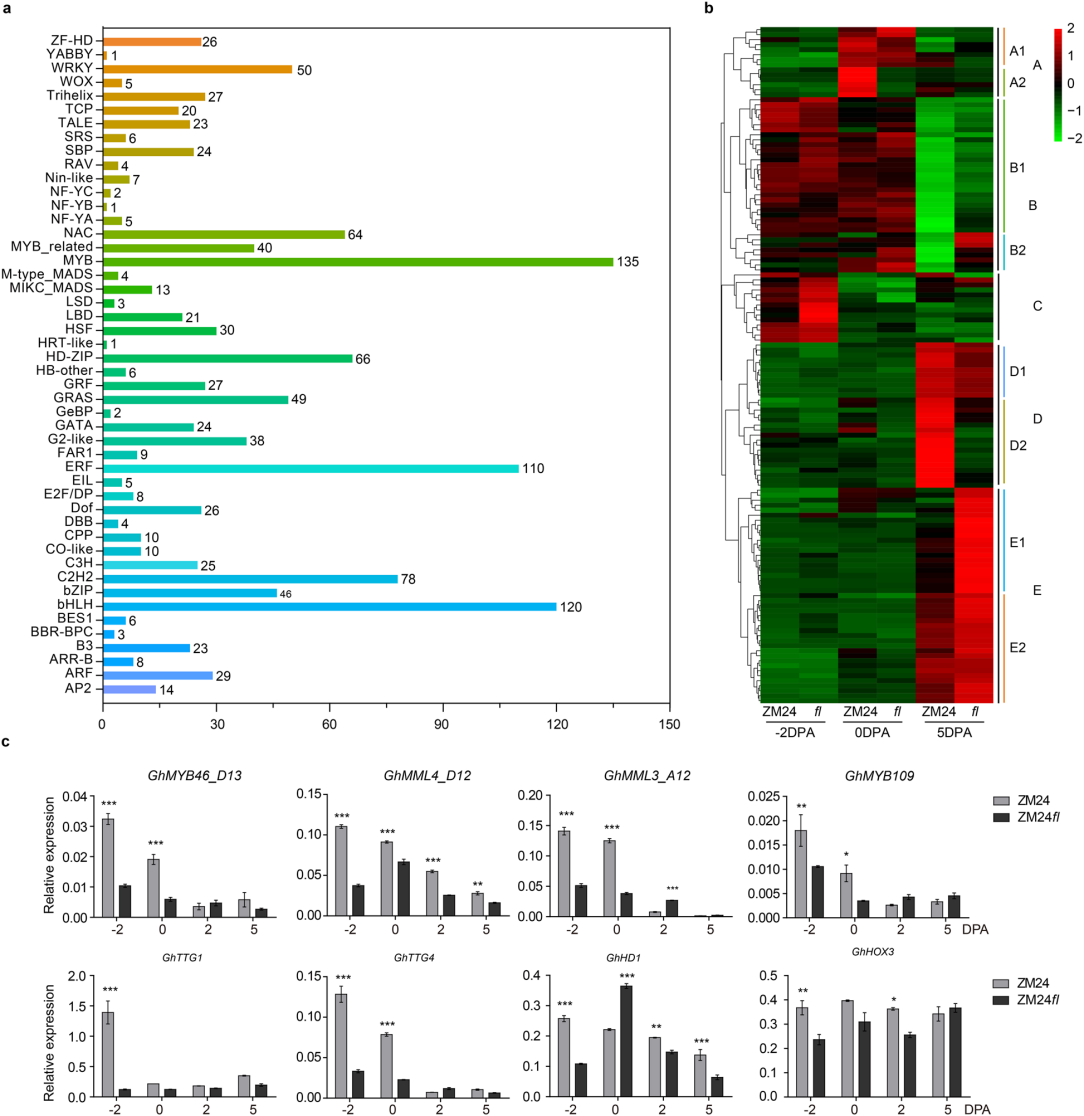
Transcription factors (TFs) identification and analysis in DEGs. **a** The classifications of the 1,258 TFs. **b** Hierarchical clustering heatmap of the 135 MYB encoding genes, which were grouped into five clusters according to their expression profile. **c** qRT-PCR of the eight genes encoding different TFs that have been documented to affect fiber initiation and elongation. The *GhHistone3* (AF024716) gene was used as reference gene, and data were shown as mean ± SD. The student’s *t*-test was used for the significance statistic

Transcription factors (TFs) play vital roles in gene expression regulation and plant development. A considerable number of researches have shown that some TFs play key roles in fiber initiation and elongation [16, 29, 36, 49]. An analysis of TFs from the DEGs set showed that 1,258 genes encode TFs, accounting for 9.7 % (1,258/12,971) DEGs, which were classified into 48 families. The largest group of TFs was the MYB (135) family, followed by the bHLH (120), ERF (110), C2H2 (78), and HD-ZIP (66) families, etc. (Fig. 5a). Of these differentially expressed TFs, 90 (86 up- and 4 down-regulated), 27 (1 up- and 26 down-regulated), and 279 (179 up- and 99 down-regulated) TFs were identified comparing *fl* with ZM24 at -2, 0, 5 DPA, respectively (Additional file 4 and 6). In horizontal comparison, 210 TFs were differentially expressed in the comparison of “*fl*_-2 vs *fl*_0”, of which the most DE-TFs members were ERF (55) and bHLH (24). Two hundred and two TFs including MYB (27), MYB_related (19), ERFs (18), etc. were identified in 0 DPA compared to -2 DPA in ZM24 ovules. Correspondingly, the number of DE-TFs between 0 vs 5 DPA has reached 397 and 842 in *fl* and ZM24, respectively. In *fl*, the most differentially expressed TFs include MYB (52), NAC (48), HD-ZIP (33), C2H2 (30), bHLH (29), ERF (25), WRKY (23), etc., whereas, the TFs groups of bHLH (83), MYB (82), ERF (52), HD-ZIP (51), C2H2 (50) were the most differentially expressed transcription factor families in ZM24 (Additional file 4).

Given that MYB TFs play key roles in fiber initiation, hierarchical clustering analysis was performed to show the expression profiles of the 135 MYB TFs encoding genes in two lines (Fig. 5b). These MYBs were grouped into 5 main clusters (A to E). The clusters C and A harbored 14 TFs each and genes in them are specifically expressed in -2 and 0 DPA, respectively. Furthermore, 6 genes in A2 were showed higher expression in ZM24 but low expression in *fl* on 0 DPA. Twenty-seven genes clustered in B1 were highly expressed in -2 and 0 DPA, whereas the other 8 MYB TFs in B2 showed high expression in all samples except at 5 DPA of ZM24. In clusters D and E, 11 genes in D1 and 22 genes in E2 were specifically expressed in 5 DPA. In addition, 18 *MYB*s in group D2 showed highest expression at 5 DPA of ZM24, with the remaining 21 *MYB*s clustered in E1 showed the highest expression at 5 DPA of *fl*. Several known positive MYB genes that have been reported to regulate fiber initiation [16, 19, 29, 49] were selected for the further qPCR experiment, and most of which showed a down-regulated profile in *fl* (Fig. 5c). In addition, two HD-ZIP TFs, GhHD1, and GhHOX3 that promoted fiber initiation and elongation by mediating accumulation of ethylene and reactive oxygen together [40, 50] were down-regulated in *fl* at -2 DPA, 2 DPA, and 5 DPA. Furthermore, two TRANSPARENT TESTA GLABRA loci (TTG1 and TTG4) that were positive to fiber initiation also showed lower expression in *fl* than that of ZM24 during fiber initiation [18, 56]. These results indicated that different MYBs and other TFs might exert specific roles during the different fiber developmental stages, which help to the identification of distinct TFs and the detailed gene transcription regulation mechanisms underlying the fiber initiation and primary development.

### Functions analysis of DELs based on lncRNA-mRNA co-expression network

**Fig. 6:**
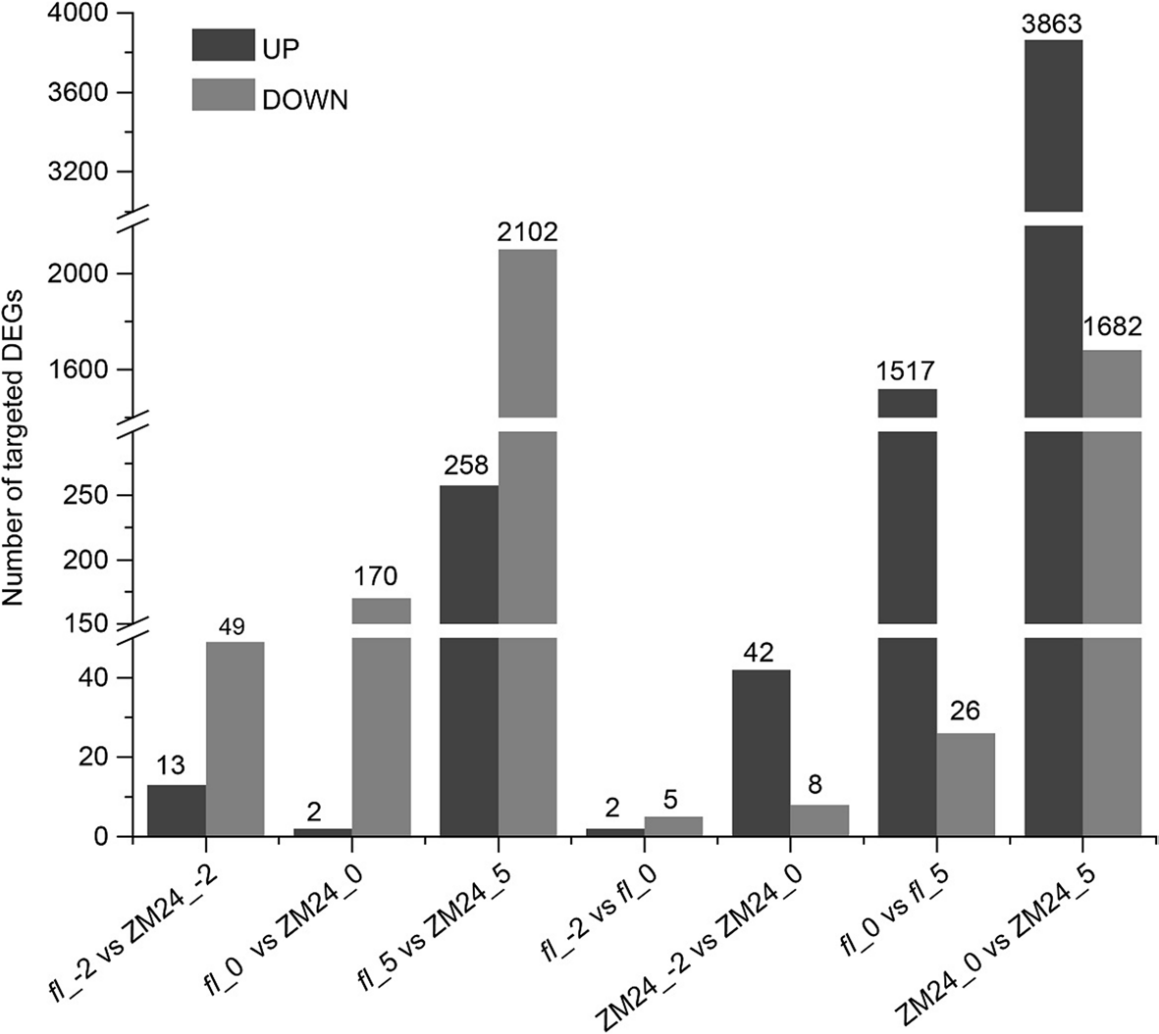
Number of up- and down-regulated targeted genes of DELs in horizontal and vertical comparisons between *fl* and ZM24. Seven comparisons were established and the differentially expressed lncRNAs were identified and summarized

To further explore the roles of DELs, cis-targeted genes and trans-targeted genes of DELs were identified by calculating PCC values and distances between lncRNAs and mRNAs. Finally, 249 DELs were co-expressed with 5,501 DE_mRNAs. Thereby 70,987 pairs of lncRNA-mRNAs were identified in all the comparisons (Additional file 7). According to the expression profile, the targeted DEGs were summarized as shown in Fig. 6. In ovules of -2, 0, and 5 DPA, 49, 170, and 2,102 targeted mRNAs were down-regulated in *fl* compared with ZM24. In the comparison of “-2 DPA vs 0 DPA”, 7 (2 up, 5 down) and 50 (42 up, 8 down) DE-mRNAs were identified in *fl* and ZM24, respectively. Similarly, 1,543 (1,517 up, 26 down) and 5,545 (3,863 up, 1,682 down) DE-mRNAs were identified in “*fl*_0 vs *fl*_5” and “ZM24_0 vs ZM24_5”, respectively. Thus, the KEGG enrichment of targeted DE-mRNAs was performed to investigate the functions of DELs. As a result, of the down-regulated targeted mRNAs in *fl* related to ZM24, genes were significantly enriched in fatty acid elongation and lipid metabolism at 0 and 5 DPA. Of the up-regulated targeted mRNAs, DE-mRNAs at 5 DPA related to 0DPA in *fl* were mainly enriched in amino acid metabolism, phenylpropanoid biosynthesis, fatty acid degradation and flavonoid biosynthesis pathways; however, the pathways of fatty acid elongation, lipid metabolism, carbohydrate metabolism, energy metabolism were enriched by DE-mRNAs of that in ZM24 (Additional file 8).

**Fig. 7:**
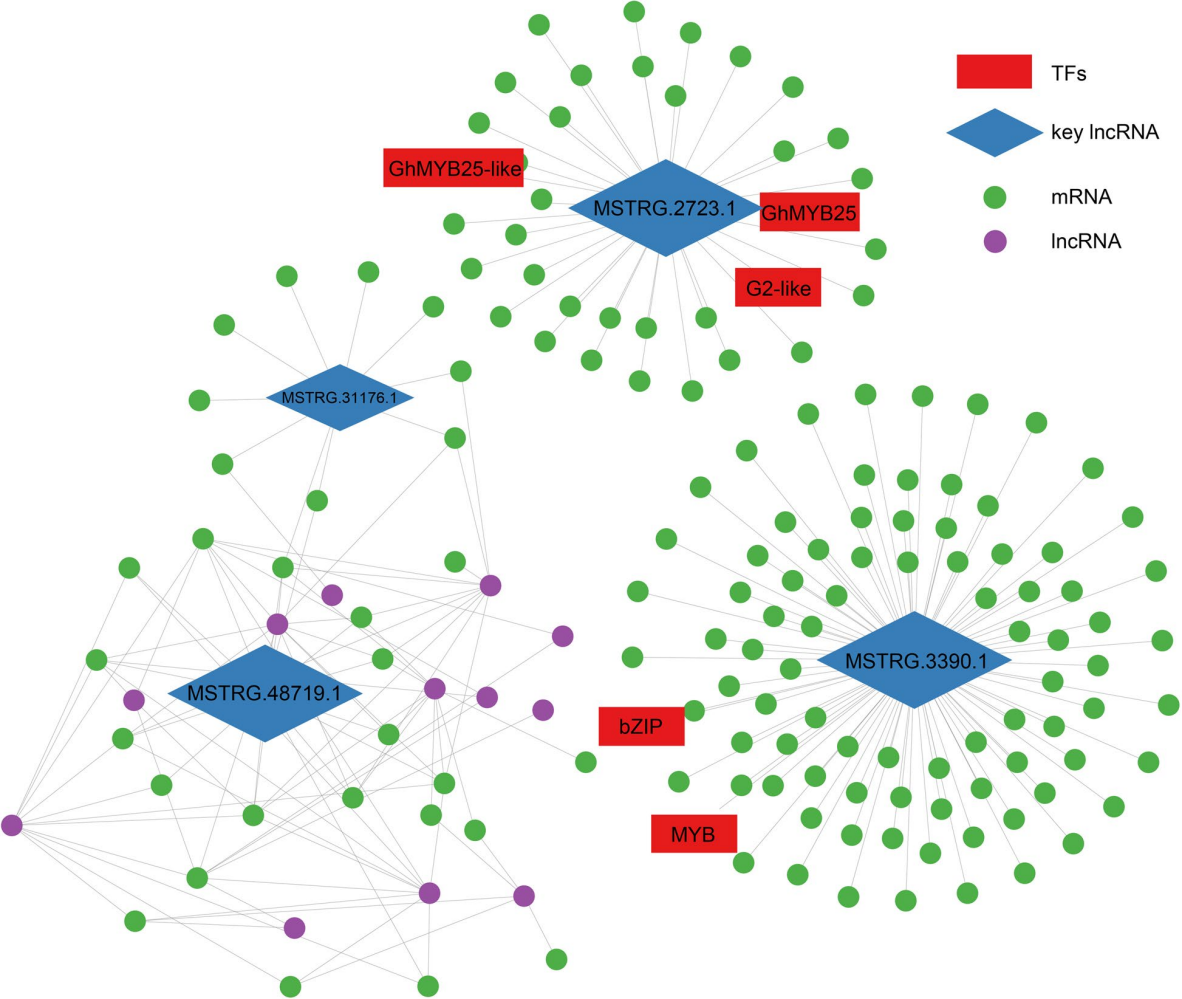
The co-expression network between DELs, the targeted DE-mRNA and TFs. Four lncRNAs showing significant expression difference between ZM24 and *fl* were identified and presented here. The blue rectangle and rhombus indicate the TFs and potential associated lncRNA. Other targeted mRNA and lncRNA were shown with green and yellow circles. The network was visualized by Cytoscape 3.6.1

To comprehensively visualize the relationship between lncRNAs and the targeted mRNAs, the co-expression network among DELs, DE-mRNAs, and TFs at 0 DPA were shown using Cytoscape software (Fig. 7). The center lncRNAs MSTRG.2723.1 and MSTRG.3390.1 are more important and independent lncRNAs that regulate many gene transcriptions including some known key TFs coding genes (e.g., *MYB25*, *MYB25-Like*, *bZIP*) in fiber initiation. Other types of lncRNAs such as MSTRG.31176.1 and MSTRG.48719.1 regulate genes transcription reciprocally, in which other different lncRNAs are also involved. Above these imply the diversity and complexity of molecular mechanisms by lncRNAs and provide a possible lncRNA-TFs regulation model in the fiber cell initiation.

## Discussion

With the glabrous mutant of cotton and high-throughput technology, generous coding-and non-coding RNAs and associated mechanisms have been identified in the fiber development including TFs, miRNAs and lncRNAs and so on [14, 28, 51, 69]. Many studies on non-coding RNAs in cotton have been limited to small RNAs until now. For instance, a lot of miRNAs specifically expressed during anther development or callus were identified in male sterile cotton as well as cotton somatic embryogenesis [57, 64]. Gong *et al*. revealed the 33 conserved miRNAs families between the A and D genomes [9]. On the genomic level, the expression of 79 miRNAs families was studied and 257 novel miRNAs were identified related to cotton fiber elongation [63]. In addition, two key miR828 and miR858 were proved the roles in the regulation of homoeologous *MYB2* (*GhMYB2A* and *GhMYB2D*) in *G. hirsutum* fiber development [11].

As a kind of long non-coding RNA, lncRNA provides more regulatory mechanisms for gene expression, protein synthesis, chromatin remodeling etc., while it is not clear about the detailed lncRNAs and the underlying mechanism in fiber development. A previous study identified 30,550 lincRNAs loci and 4,718 lncNATs loci, which are rich in repetitive sequences and preferentially expressed in a tissue-specific manner with weak evolutionary conservation. Further, lncRNAs showed overall higher methylation levels, and their expression was less affected by gene body methylation [52]. Using the epidermal cells from the ovules at 0 and 5 DPA from Xu142 and Xu142*fl*, 35,802 lncRNAs and 2,262 circular RNAs (circRNAs) were identified, of which 645 lncRNAs were preferentially expressed in the fibreless mutant Xu142*fl* and 651 lncRNAs were preferentially expressed in the fiber-attached lines; three lncRNAs XLOC_545639, XLOC_039050, and XLOC_079089 all showed the solid function in fiber development by VIGS assay [14]. Here, a novel glabrous mutant-ZM24*fl*, which showed excellent somatic embryogenesis induction was used to identify the key lncRNA involved in fiber initiation development [59].

Totally, 3,288 lncRNA transcripts were identified from the -2 DPA, 0 DPA and 5 DPA ovules of ZM24 and *fl*, which is significantly different from the number of identified lncRNA in Xu142*fl* [14] and *G*. *barbadense* L. cv 3-79 [52]. To identify the causal lncRNAs for fiber initiation, some comparisons were built to analyze the differentially expressed genes including lncRNAs and mRNAs during fiber initiation and earlier elongation. The identified DELs and DEGs in comparisons of 0 DPA vs -2 DPA and 5 DPA vs 0 DPA of ZM24 and *fl* indicated that many lncRNAs and coding genes are involved in the fiber initiation and primary development, while few lncRNAs and coding genes may involve the ovule development. The analysis of the DEGs further showed that fatty acid metabolism, very long strain fatty acid synthesis and sugar metabolism play important roles in the fiber initiation of ZM24, supporting the previous results [15, 39]. Moreover, some MYB family, bHLH type TFs encoding genes were also identified the important roles in fiber initiation, which is in agreement with the function of these TFs in previous research [10, 16, 29, 36, 40, 49, 50]. To uncover the upstream factors such as lncRNAs, we focused on the comparisons of ZM24_0 DPA vs *fl*_0 DPA and ZM24_0 DPA vs ZM24_-2 DPA to find the common lncRNAs which should be a key regulator for fiber initiation. Consequently, one lncRNA MSTRG 2723.1 was obtained, which locates on the A02G (84218766—84219942) encoding a lncNAT and covering the most coding region and partial 3’-terminal untranslated region of *Ghicr24_A02G147600* (Figure S4). The co-expression analysis further identified its potent targets including 3-ketoacyl-CoA synthase, MYB family proteins, phosphatase 2C family proteins, pectin lysase, and some uncharacterized proteins, which may are involved in fiber initiation through fatty acid pathway, cell wall plasticity, MYB-mediated signaling etc. These results provide important clues for the upstream regulatory lncRNAs in fiber initiation and novel information associated with the fiber development regulation network. In addition, MSTRG 3390.1, MSTRG 48719.1, and MSTRG 31176.1 were also identified some positive correlation between fiber development and ovule development. The sequence analysis indicated that these lncRNAs are different from the previous lncRNAs XLOC_545639, XLOC_039050, and XLOC_079089 [14]. The target analysis also implied the possible interaction between different lncRNAs through mediating the common targets, which provide novel clues to explore the regulatory lncRNAs and underlying mechanisms in fiber development. Even with some achievement of lncRNAs, the understanding of the underlying mechanism of lncRNAs regulating targets or chromosome remodeling still needs more work to disclose.

## Conclusion

Here, a novel glabrous cotton mutant ZM24*fl* was identified and applied to study the potential lncRNAs for fiber development with high-throughput sequencing. ZM24*fl* is derived from an elite cultivar of ZM24, which posses high callus induction and somatic embryogenesis ability, and is endowed with the valuable receptor for cotton genetic transformation [59]. Through the RNA-Seq and analysis in different ovules of ZM24 and *fl*, 3,288 lncRNAs were identified and some differentially expressed lncRNAs responsible for fiber (lint and fuzz) initiation and fiber earlier elongation were showed. Collectively, four lncRNAs MSTRG.2723.1, MSTRG.3390.1, MSTRG.48719.1 and MSTRG.31176.1 were showed potential important roles in fiber development, and the analysis of the target implied that MSTRG 2723.1 may function upstream of fatty acid metabolism, MBY25-mediating pathway, and pectin metabolism to regulate fiber initiation; the co-expression analysis between lncRNAs and targets further indicated the distinct models of different lncRNAs and interaction between lncRNAs, which provide precious information for illumination of the molecular mechanism of lncRNAs in fiber development of cotton.

## Materials and methods

### Plant Materials

*Gossypium hirsutum L*. acc. Zhongmiansuo24 (ZM24) and a natural fuzzless-lintless (*fl*) mutant from ZM24 were used and grown under standard field conditions in the Institute of Cotton Research of the Chinese Academy of Agricultural Sciences (Zhengzhou research base, Henan). The ovule tissues were collected from cotton bolls on -2, 0, and 5 DPA using a sterile knife. All materials were frozen in liquid nitrogen immediately and stored at -80 °C for the following experiments.

### Microscopic observation of fiber initiation on ovules epidermis

To study the fiber initiation phenotypes of ZM24 and *fl*, the cotton bolls of two lines on -2, 0, 1 and 2 DPA were collected. Then, the ovules were stripped from the bolls in the middle region. Immediate Scanning electron microscopy (Hitachi) was performed to observe the ovule epidermis as described previously [14].

### Strand specific libraries construction and sequencing

Total RNAs of each ovule sample was extracted using the RNAprep Pure Plant Kit (Tiangen, Beijing, China) following the manufacturer’s instruction. Total RNAs of each sample was quantified and qualified by Agilent 2100 Bio-analyzer (Agilent Technologies, Palo Alto, CA, USA), Nanodrop 2000 (Thermo Fisher Scientific Inc.), and 1% agarose gel. RNA with RIN value above 7 was used for following library construction. The rRNA was removed using the Ribo-Zero™ rRNA removal Kit. The ribosomal depleted RNA was then used for sequencing library preparation according to the manufacturer’s protocol (NEBNext® Ultra™ Directional RNA Library Prep Kit for Illumina®). The cDNA libraries with different indices were multiplexed and loaded on an Illumina Hiseq2500 with 150 base pair (bp) paired-end (PE150) raw reads according to the manufacturer’s instruction (Illumina, San Diego, CA, USA). RNA-Seq raw data with accession number SRP285346 was uploaded in the NCBI sequence read archive (http://www.ncbi.nlm.nih.gov/sra/) and the accession numbers of the twenty-four runs are SRR12710181-SRR12710192, and SRR12718970-SRR12718981.

### Mapping to the reference genome and LncRNAs identification

The raw data in fastq format were filtered with cutadapter (v1.9.1) software [30]. Clean data were obtained by removing reads that contained adapter, poly-N and base with Phred quality < 20 in 3’ or 5’ end, and the reads of length < 75 bp were removed after filtering. Finally, the GC percentage and Q30 of each sample were calculated using FastQC software (https://www.babraham.ac.uk/) and shown in Table S1. Clean data were mapped to the ZM24 genome (https://github.com/gitmalm/Genome-data-of-*Gossypium*-*hirsutum*/) [66] using HISAT(v2.1.0) [20, 21] software with the parameter “--rna-strandness RF”. Transcriptomes of each sample were assembled based on mapped reads and were merged by StringTie software (v2.0) [34, 35]. Transcripts annotation was performed using Cuffcompare [47]. Long non-coding RNA was identified as following steps: 1) transcripts with class codes of “i”, “u”, “x”, “j” representing the intronic transcripts, long intergenic noncoding RNAs (lincRNAs), long noncoding natural antisense transcripts (lncNAT), and the sense transcripts, respectively, were selected. 2) Transcripts with length > 200 bp, coverage > 1, FPKM > 0.5; 3) The CNCI [44], CPC [22] and PfamScan software were used to assessed protein-coding ability [7], with the parameter of (CPC score < 0, CNCI score < 0).

### Differential expression analysis

The FPKM values and counts of genes and lncRNAs in each sample were calculated using StringTie and Ballgon [35]. Differential expression analyses were conducted by edgeR in R package [37, 38]. The DEGs and DELs were identified with an expression FPKM > 1.0, FDR (false discovery rate < 0.001), and |log2( fold change value)| ≥1 between each pairwise comparison.

### Co-expression analysis between lncRNA and mRNA

To unveil the potential functions of DELs between the two genotypes, two interaction models of lncRNAs and protein-coding genes (lncRNAs/PC-genes) including cis- and trans-target were analyzed: 1) the Pearson correlation coefficient (PCC) between differentially expressed lncRNAs and mRNAs were calculated using the OmicShare tools (https://www.omicshare.com/) with the expression profiles (FPKM). The lncRNA-mRNA pairs with |PCC| > 0.95 and *p*-value < 0.01 were regarded as trans interaction between lncRNAs and mRNAs. 2) Protein-coding genes with a distance less than 20 kb from the upstream or downstream of lncRNAs were putative cis interaction. The co-expression networks were visualized by Cytoscape 3.6.1 [41].

### GO and KEGG

To explore the functions of DEGs and lncRNAs between ZM24 and *fl*, the gene ontology (GO) enrichment was performed using the BLASTP program [1] and GO databases (http://archive.geneontology.org/latest-lite/) and (http://ftp.ncbi.nlm.nih.gov/gene/DATA/). Kyoto Encyclopedia of Genes and Genomes (KEGG) enrichment analysis was performed at KOBAS 3.0 website [58, 61] (http://kobas.cbi.pku.edu.cn/kobas3).

### Q -PCR analysis

Ovules from bolls at -2, 0, 2, and 5 DPA were collected, and then total RNAs were extracted using the RNAprep Pure Plant Kit (Polysaccharides & Polyphenolics-rich, Tiangen, Beijing, China) following the manufacturer’s instruction. Each reverse-transcribed reaction was performed with 1 μg RNA using a transScript® First-Strand cDNA Synthesis SuperMix (AT301-02, TransGen). The real-time PCR was performed on Roche 480 PCR system with a SYBR-Green Real-time PCR SuperMix (AQ101-01, TransGen). The 20 uL reaction volumes in each well contain 1 μL cDNA, 8.2 μL sterile water, 10 μL Mix, and 0.4 μL each of the forward and reverse primers. The Q-PCR procedures were as: pre-incubation of 30 s at 95 °C; followed by denaturation at 95 °C for 10 s, primer annealing at 55 °C for 10 s, and then extension at 72 °C for 30 s; finally, a melting curve at 95 °C for 30 s to check the primer specificity. The *GhHistone3* (AF024716) gene was used as a reference gene. The 2^-∆Ct^ method was used to calculate the relative expression of each gene, with three technical repetitions and three biological repetitions. Data were shown as mean ± SD. The student’s *t*-test was used for the significance statistic. The primer sequences used in the presented study are listed in Additional file 9.

## Supplementary Information

**Additional file 1.** RNA-Seq data for 12 samples

**Additional file 2.** List of lncRNAs identified from ovules of two lines during fiber initiation stage.

**Additional file 3.** Differentially expressed lncRNAs in different comparisons.

**Additional file 4.** Differentially expressed genes in different comparisons.

**Additional file 5.** The targeted genes by lncRNA MSTRG.2723.1.

**Additional file 6.** Transcription factors identification in the 12,971 DEGs.

**Additional file 7.** Differentially expressed lncRNA and their targets in all comparisons.

**Additional file 8.** The KEGG pathways of differentially expressed targeted genes of DELs.

**Additional file 9.** List of primers used in this research.

**Additional file 10: Figure S1.** Observation and comparison of *fl* and ZM24 in the different developmental stages and tissues. **a** and **f** The plants architecture of *fl* and ZM24; **b** and **c** The magnifications of white rectangular dotted bolls at 20 DPA from **a** and **f**, respectively. **d** The size comparison of bolls at 15 DPA from *fl* (left) and ZM24 (right). **e** The size and shape of leaves from two lines *fl* (left) and ZM24 (right). **g** and **i**The epidermal hair on the abaxial leaf surface of *fl* and ZM24; (h) The stem epidermal hair of *fl* (up) and ZM24 (down); bars in **a**-**f**: 2.0 cm; bars in **g**-**i**: 1000 μm.

**Additional file 11: Figure S2.** Heatmap shows the Pearson correlation coefficients among the 12 samples.

**Additional file 12: Figure S3.** Gene ontology classifications of DEGs in ovules of ZM24 vs *fl* at 5 PDA. The most highly enriched GO terms showed the 1,378 down- and 2,608 up-regulated genes in ovules of ZM24 vs *fl* at 5 DPA.

**Additional file 13: Figure S4.** The physical location of lncRNA MSTRG.2723.1 on the ZM24 genome. The MSTRG.2723.1 is a natural antisense transcript and overlaps with the gene of *Ghicr24_A02G147600*. Blue and orange rectangles represent exons and introns, respectively. Arrows indicate the direction of transcription.

## Data Availability

All the related data and files are presented including the sequences of the primers used in the Q-PCR. RNA-Seq raw data with accession number SRP285346 was uploaded in the NCBI sequence read archive (http://www.ncbi.nlm.nih.gov/sra/) and can be accessible with bioproject archive number PRJNA665585 (http://www.ncbi.nlm.nih.gov/bioproject/).
