# Supplementary figures and images for: RNA-Seq with a novel glabrous-ZM24fl reveals some key lncRNAs and the associated targets in fiber initiation of cotton
Source: BMC Plant Biol. 2022 Feb 3;22:61. doi: 10.1186/s12870-022-03444-9 (PMC8815142; doi:10.1186/s12870-022-03444-9)

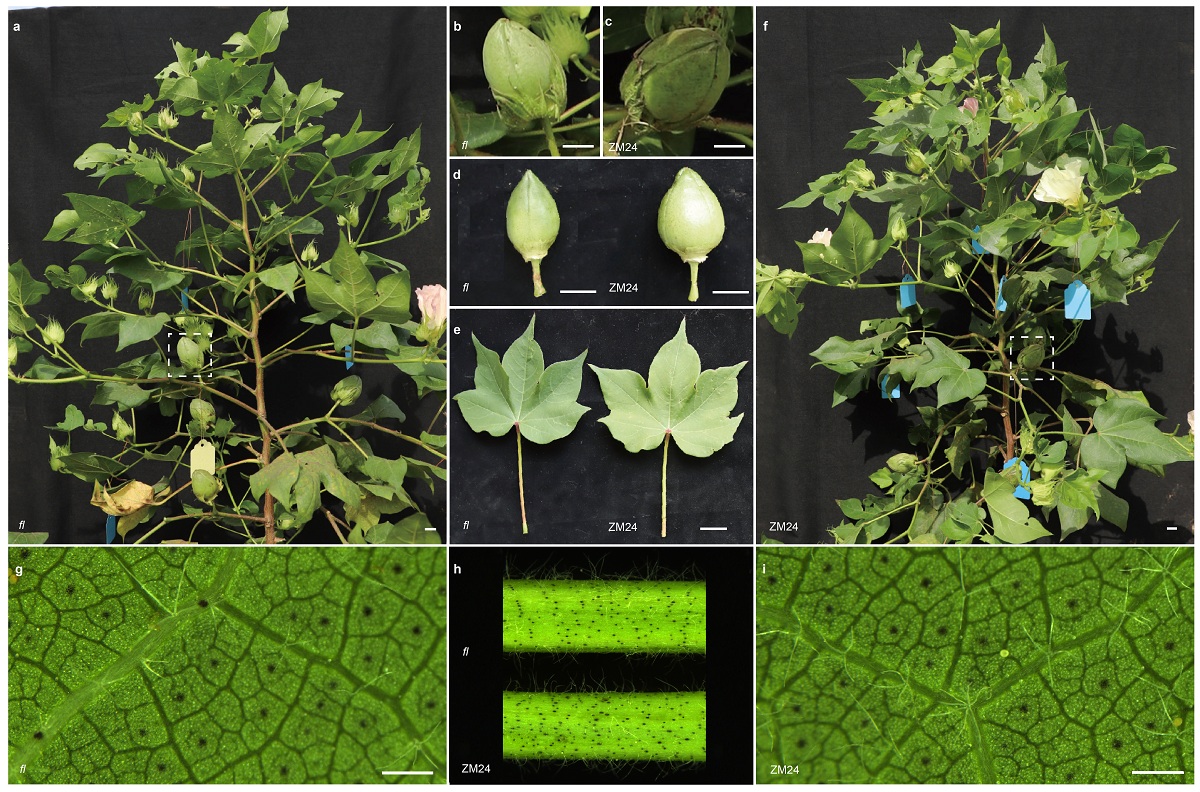

Supplement: Supplementary file 10 — Additional file 10: Figure S1. Observation and comparison of fl and ZM24 in the different developmental stages and tissues. a and f The plants architecture of fl and ZM24; b and c The magnifications of white rectangular dotted bolls at 20 DPA from a and f, respectively. d The size comparison of bolls at 15 DPA from fl (left) and ZM24 (right). e The size and shape of leaves from two lines fl (left) and ZM24 (right). g and iThe epidermal hair on the abaxial leaf surface of fl and ZM24; (h) The stem epidermal hair of fl (up) and ZM24 (down); bars in a-f: 2.0 cm; bars in g-i: 1000 μm. [file 12870_2022_3444_MOESM10_ESM.jpg]

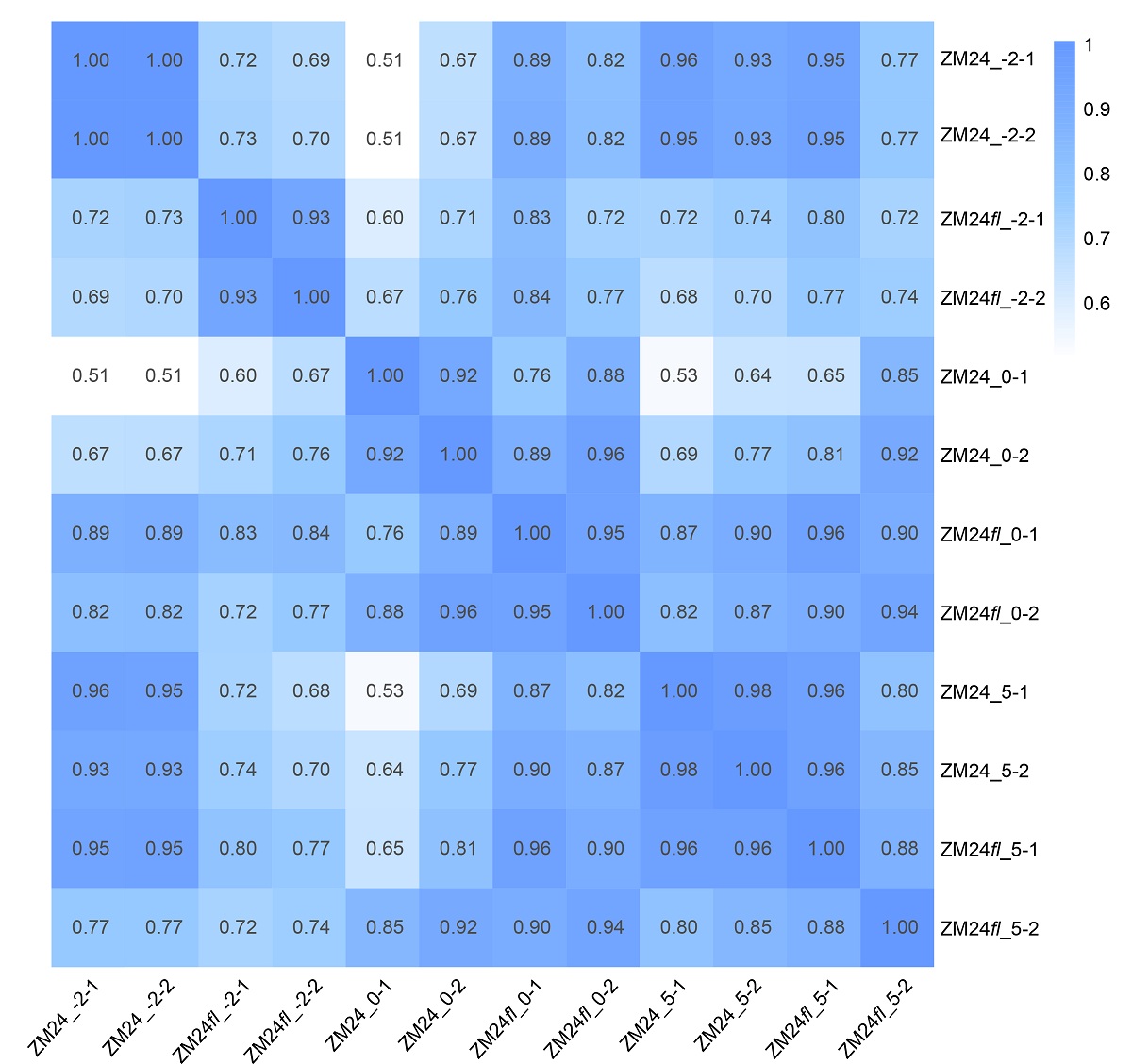

Supplement: Supplementary file 11 — Additional file 11: Figure S2. Heatmap shows the Pearson correlation coefficients among the 12 samples. [file 12870_2022_3444_MOESM11_ESM.jpg]

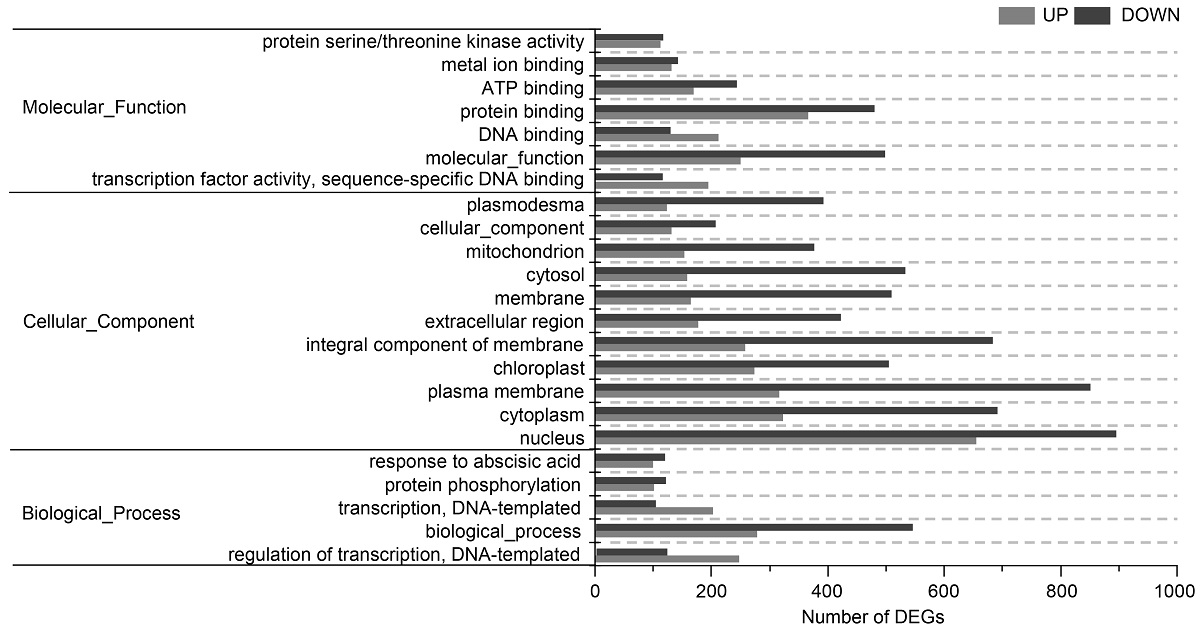

Supplement: Supplementary file 12 — Additional file 12: Figure S3. Gene ontology classifications of DEGs in ovules of ZM24 vs fl at 5 PDA. The most highly enriched GO terms showed the 1,378 down- and 2,608 up-regulated genes in ovules of ZM24 vs fl at 5 DPA. [file 12870_2022_3444_MOESM12_ESM.jpg]

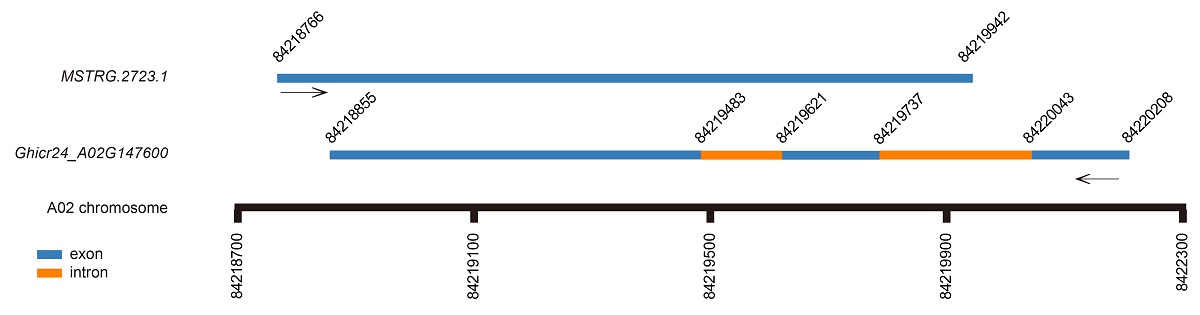

Supplement: Supplementary file 13 — Additional file 13: Figure S4. The physical location of lncRNA MSTRG.2723.1 on the ZM24 genome. The MSTRG.2723.1 is a natural antisense transcript and overlaps with the gene of Ghicr24_A02G147600. Blue and orange rectangles represent exons and introns, respectively. Arrows indicate the direction of transcription. [file 12870_2022_3444_MOESM13_ESM.jpg]
